# *Beauveria bassiana* interacts with gut and hemocytes to manipulate* Aedes aegypti* immunity

**DOI:** 10.1186/s13071-023-05655-x

**Published:** 2023-01-17

**Authors:** Ricardo de Oliveira Barbosa Bitencourt, Thaís Almeida Corrêa, Jacenir Santos-Mallet, Huarrison Azevedo Santos, Carl Lowenberger, Haika Victória Sales Moreira, Patrícia Silva Gôlo, Vânia Rita Elias Pinheiro Bittencourt, Isabele da Costa Angelo

**Affiliations:** 1 Graduate Program in Veterinary Sciences, Veterinary Institute, Federal Rural University of Rio de Janeiro, Seropédica, RJ Brazil; 2 Oswaldo Cruz Foundation, IOC-FIOCRUZ-RJ, Rio de Janeiro, RJ Brazil; 3 Centre for Cell Biology, Development and Disease, Department of Biological Sciences, Simon Fraser University, Burnaby, BC V5A 1S6 Canada; 4 Department of Animal Parasitology, Veterinary Institute, Federal Rural University of Rio de Janeiro, Seropédica, RJ Brazil; 5 Department of Epidemiology and Public Health, Veterinary Institute, Federal Rural University of Rio de Janeiro, Seropédica, RJ Brazil; 6 FIOCRUZ-PI, Teresina, Piauí Brazil; 7 Iguaçu University-UNIG, Nova Iguaçu, RJ Brazil

**Keywords:** Biological control, Entomopathogenic fungi, Hemocytes, Antimicrobial peptides, Mosquito, Immune system

## Abstract

**Background:**

Mosquito-borne diseases affect millions of people. Chemical insecticides are currently employed against mosquitoes. However, many cases of insecticide resistance have been reported. Entomopathogenic fungi (EPF) have demonstrated potential as a bioinsecticide. Here, we assessed the invasion of the EPF *Beauveria bassiana* into *Aedes aegypti* larvae and changes in the activity of phenoloxidase (PO) as a proxy for the general activation of the insect innate immune system. In addition, other cellular and humoral responses were evaluated.

**Methods:**

Larvae were exposed to blastospores or conidia of *B. bassiana* CG 206. After 24 and 48 h, scanning electron microscopy (SEM) was conducted on the larvae. The hemolymph was collected to determine changes in total hemocyte concentration (THC), the dynamics of hemocytes, and to observe hemocyte-fungus interactions. In addition, the larvae were macerated to assess the activity of PO using L-DOPA conversion, and the expression of antimicrobial peptides (AMPs) was measured using quantitative Real-Time PCR.

**Results:**

Propagules invaded mosquitoes through the midgut, and blastopores were detected inside the hemocoel. Both propagules decreased the THC regardless of the time. By 24 h after exposure to conidia the percentage of granulocytes and oenocytoids increased while the prohemocytes decreased. By 48 h, the oenocytoid percentage increased significantly (*P* < 0.05) in larvae exposed to blastospores; however, the other hemocyte types did not change significantly. Regardless of the time, SEM revealed hemocytes adhering to, and nodulating, blastospores. For the larvae exposed to conidia, these interactions were observed only at 48 h. Irrespective of the propagule, the PO activity increased only at 48 h. At 24 h, *cathepsin B* was upregulated by infection with conidia, whereas both propagules resulted in a downregulation of *cecropin* and *defensin A*. At 48 h, blastospores and conidia increased the expression of *defensin A* suggesting this may be an essential AMP against EPF.

**Conclusion:**

By 24 h, *B. bassiana* CG 206 occluded the midgut, reduced THC, did not stimulate PO activity, and downregulated AMP expression in larvae, all of which allowed the fungus to impair the larvae to facilitate infection. Our data reports a complex interplay between *Ae. aegypti* larvae and *B. bassiana* CG 206 demonstrating how this fungus can infect, affect, and kill *Ae. aegypti* larvae.

**Graphical Abstract:**

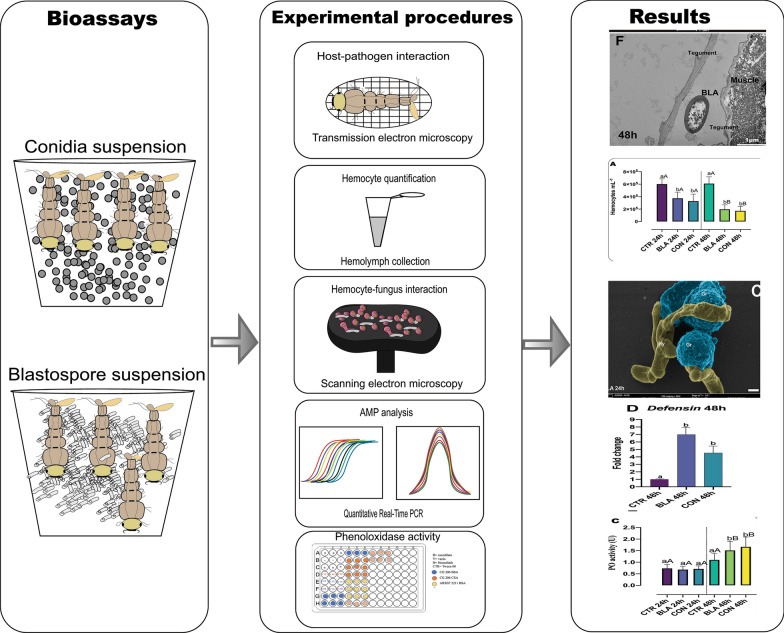

**Supplementary Information:**

The online version contains supplementary material available at 10.1186/s13071-023-05655-x.

## Background

Mosquitoes transmit many arboviruses [1]. In particular, *Aedes aegypti* (Diptera: Culicidae) transmits dengue (DENV), chikungunya (CHIKV), yellow fever (YF) and Zika (ZIKV) viruses that causes morbidity and mortality in subtropical and tropical countries, including Brazil [2]. Classical mosquito control methods are based on chemical insecticides and integrated pest management practices to eliminate larval habitats [3]. However, there are many reports of insecticide resistance developed by *Ae. aegypti* due to the overuse of chemical insecticides [4–9]. Therefore, biological controls might represent a complementary tool for controlling *Aedes* sp.

*Beauveria bassiana* (Bals.) Vuill. (Hypocreales: Cordycipitaceae) is an entomopathogenic fungus (EPF) with the potential to control mosquitoes of public health concern [10–15]. The EPF infection process starts with the attachment by conidia and germ tube development. To overcome the tegument, the fungus uses a combination of mechanical pressure and an array of cuticle-degrading enzymes [16]. The following steps include blastospore proliferation, toxin production, and hyphal development that take up the energy resource from the insect. After exhausting the hosts’ nutrients, conidiogenesis, the production of new conidia, occurs on the cadaver [17].

Conidia and blastospores (similar to hyphal bodies) of EPF have been tested to control *Ae. aegypti* [10, 14, 15, 18]. However, both are distinct in several ways [19]. Conidia are terrestrial spores with hydrophobic walls and are generally more resistant to desiccation than blastospores [16]. On the other hand, blastospores are polymorphic yeast-like cells produced within the insect hemocoel. They have a thin cell wall without ß-glucans to act as camouflage against the immune system of the insect hosts.

Insects rely on their physical barriers (e.g., tegument and epithelial cells of the gut), as well as cellular and humoral components of their innate immune system to protect themselves from invading fungi and other microorganisms [20–22]. The mosquito’s tegument is a complex structure with layers (epicuticle, exo-cuticle, endo-cuticle, and epidermis) covered by a waxy coating [23]. The tegument has many functions, such as protecting the mosquitoes against microbial attack [23, 24]. Enterocytes are epithelial cells from the mosquito gut [25] responsible for producing the peritrophic membrane, absorbing nutrients, and protecting against parasites [25, 26]

The cellular immune response, especially in the hemocoel, is mediated by hemocytes that play a pivotal role in phagocytosis, nodulation, and encapsulation processes [27]. Hemocytes mediate humoral, and melanization processes after pathogens are recognized [27, 28]. Six types of hemocytes have been reported in *Ae. aegypti* [29]. However, granulocytes and plasmatocytes are primarily involved with phagocytosis and encapsulation while oenocytoids are involved in nodulation and melanization [30].

The humoral immune response is responsible for responding to the presence of microbes and acts principally through the Toll, IMD, and JAK-STAT molecular pathways, which ultimately results in the expression of potent antimicrobial peptides (AMPs). AMPs create pores, depolarize microbial membranes, and may affect microbial replication [22, 31–33]. Initially, the Toll pathway was reported to act against Gram-positive bacteria and fungi and the IMD pathway against Gram-negative bacteria, but there is a growing literature on the cross talk and among and between immune pathways and the co-regulation of AMP expression through multiple pathways [34–37]. In addition, the JAK-STAT and RNAi pathways are generally regarded as antiviral immune pathways [31–33].

Efforts to eliminate the EPF in insects involve coordinated responses to phagocytose fungal bodies, to express AMPs, and to encapsulate and melanize fungal bodies through the melanization cascade [38] that relies on the activity of multiple enzymes that involve the conversion of the inactive precursor prophenoloxidase (PPO) to the active phenoloxidase (PO) that ultimately produces the melanin that kills the fungi [39].

*Beauveria bassiana* CG 206 has demonstrated potential as a larvicide against *Ae. aegypti* [10]. Here, the impact of *B. bassiana* CG 206 blastospores or conidia against *Ae. aegypti* larvae was assessed. The study focused on the route of infection and the activation of PO activity, as well as cellular and humoral responses. Our studies revealed contrasting effects in EPF infection, bringing new insights into understanding fungal-mosquito interactions.

## Methods

### Production of fungus and insects

*Beauveria bassiana* sensu lato (s.l.) CG 206 was obtained from the National Center for Genetic Resources-CENARGEN, EMBRAPA, Brazil. This fungus was chosen due to its potential to kill *Ae. aegypti* larvae [10]. The fungus was grown for 15 days on potato dextrose agar artificial medium under controlled conditions [25 ± 1 °C; relative humidity (RH) ≥ 80%]. After sporulation, the conidia were harvested and suspended in 0.03% Tween-80 sterile dechlorinated tap water solution (v/v). The suspensions were homogenized in a vortex for one minute. Blastospores were obtained after incubating conidia in 40 mL modified Adamek’s medium at 27 °C and 200 rpm (TE-424^®^, Tecnal) for 72 h [10]. Propagule concentrations were adjusted to 1 × 10^7^ propagules mL^−1^ using a hemocytometer [40].

Eggs of *Ae. aegypti* (Rockefeller strain) were provided by the Microbial Control Laboratory from the Federal Rural University of Rio de Janeiro (UFRRJ, Brazil). For larval hatching, the eggs were maintained in a tap water bowl (2 L) under controlled conditions [27 ± 1 °C; (RH) ≥ 80%] on a 12-h light/dark cycle as described previously [10]. The larvae were fed daily on pulverized fish food (0.05 g/L).

### Transmission electron microscopy

Groups of 10 s instar *Ae. aegypti* (*N* = 30) larvae (L_2_) were exposed to 10 mL of blastospore (BLA) or conidial (CON) suspensions at 1 × 10^7^ propagules mL^−1^ [10]. After 24 or 48 h, survived larvae (*N* = 3) were fixed in 2.5% (v/v) glutaraldehyde in 0.1 M sodium cacodylate pH 7.2 for 24 h, at 4 °C. The larvae were washed three times (10 min each) in 0.1 M sodium cacodylate pH 7.2 (Sigma-Aldrich©, US). The specimens were fixed in 1% osmium tetroxide (O_5_O_4_) solution at room temperature. The protocol for TEM was conducted as described previously [41].

After 60 min, the washing procedure was repeated three times, followed by a dehydration procedure by ascending ethanol series (50–90%) for 5 min and in 100% ethanol (30 min each) three times. Then, the larvae were immersed in a 100% ethanol and EPON resin solution (1:1) at − 4 °C, and after 18 h, they were incubated in pure EPON™ resin (HEXION™, Ohio, US) and polymerized for 48 h at 60 °C [41]. Finally, ultrathin sections were cut using an ultramicrotome, stained with uranyl acetate and lead; mounted onto copper grids and examined in an FEI Tecnai T20^®^ (Philips©, Amsterdam, Netherlands) and HT 7800^®^ (Hitachi©, Tokyo, Japan) transmission electron microscopes (TEM) at 120 kV.

### Total hemocyte concentration and population dynamics

In our experimental design, 30 larvae (*N* = 90) were exposed to 30 mL of blastospore or conidial suspensions at 1 × 10^7^ propagules mL^−1^. After 24 or 48 h post-exposure (p.e.), larvae were beheaded with a sterile scalpel blade. The pool of hemolymph of 10 larvae (per group) was collected with a microcapillary tube and then inoculated directly into 20 µL of L-15-Leibovitz medium (L15) (Biosera^®^, MO, USA) that was kept on ice throughout the collection [42, 43]. Ten microliters of the L15 + hemolymph were immediately placed on a hemocytometer, and the total number of hemocytes was quantified at × 400 [44].

For the analysis of hemocytes, the pool of hemolymph (*N* = 10) was collected and stained with Giemsa (Additional file 1: Text S1), and hemocytes were identified (Additional file 1: Fig. S1). Six stained slides per group were prepared and observed using a compound microscope. Two hundred hemocytes were quantified, identified, and then the numbers of cells were converted to percentages [45]. The experiments were repeated three times totalling 18 glass slides per group. For full protocol of hemolymph collection and pictures, please see the Additional file 1.

### Scanning electron microscopy (SEM) of mosquito hemocytes

One hundred larvae (*N* = 300) were exposed to 100 mL of blastospore or conidial suspensions at 1 × 10^7^ propagules mL^−1^. After 24 and 48 h, a pool of hemolymph from 100 surviving larvae per group was transferred to microtubes and fixed in glutaraldehyde (2.5%, pH 7.2) (v/v) at 4 °C overnight. The microtubes were previously treated with Sigmacote^®^ (Sigma-Aldrich©, US) to avoid the adhesion of hemocytes to the wall. Next, the microtubes were centrifuged (4 °C, 10 min, 2000 rpm) (Centrifuge 5418, Eppendorf^®^), the supernatant was discarded, and the pellet was resuspended in 100 µL of sodium cacodylate buffer (0.1 M; pH 7.2). The processes of centrifugation and resuspension were repeated three times. The samples then were fixed in 1% OsO_4_ at room temperature for 1 h [46]. After fixation, the samples were centrifugated and resuspended as previously described, followed by dehydration in an ethanol series [10]. Finally, samples were dried at the critical point in CO_2_, embedded with gold, examined, and photographed using a high-resolution SEM Auriga 40^®^ (ZEISS©, Oberkochen, Germany). Please see the Additional file 1: Fig. S2 for pictures of scanning electron microscopy of hemocytes identified.

### PO activity

Thirty larvae (*N* = 90) were exposed to EPF, as described above. After 24 or 48 h, ten surviving larvae (*N* = 30) were transferred to microtubes and crushed with 35 µL of cold PBS (0.1 M phosphate buffer, 1.5 M NaCl, pH 7.4). Samples were centrifuged for 2 min at 3000 rpm (− 4 °C) [47], and 2 µL of supernatant were inoculated into wells of a 96-well plate (KASVI^®^) and incubated on ice for 10 min with 28 µL of cacodylate buffer (0.01 M containing 0.0005 M CaCl2, pH 7.0). The samples were then incubated for 20 min with 10 µL of L-DOPA (Sigma-Aldrich^®^, US) at 4 mg/ml. Three wells containing the supernatant and cacodylate received 2 µL of trypsin (Sigma-Aldrich©, US) (1 mg/mL of supernatant) and incubated for 30 min; these were considered as 100% activity for comparison with the groups [48]. Sample absorbances were measured in an ELISA plate reader (Multiskan FC, Thermo Fisher Scientific©, Hillsboro, OR, US) at 490 nm, according to [48].

### Effect of fungal infection on AMP expression

#### RNA extraction and cDNA synthesis

Groups of 10 larvae (*N* = 30) were exposed to EPF for 24 and 48 h. Two surviving larvae (*N* = 6) were macerated, and total RNA was extracted using Trizol reagent (Invitrogen, Carlsbad, CA, US) according to the manufacturer’s recommendations. The RNA was quantified on a Qubit™ RNA High Sensitivity (HS) Assay Kits (Thermo Fisher Scientific). Reverse transcription (RT) was performed in a final volume of 20 μL, containing: 4 μL of (5X) RT Buffer, 1 μL of dNTP, 1 μL of Random Primers, 1 μL of OneScript^®^ Plus RTase, 1000 ng of RNA and nuclease-free water (DEPC). The tubes were briefly centrifuged and incubated at 50 °C for 15 min in an Eppendorf Gradient Master Cycler thermocycler (Eppendorf^®^) for cDNA synthesis at 65 °C 5 min and 42 °C for 50 min. The cDNA was diluted (1:50) with DEPC water.

#### Evaluation of AMP gene expression using Real Time Quantitative PCR (RT-qPCR)

RT-qPCR (Applied Biosystems^®^ StepOneTM Real-Time PCR Systems, ThermoFisher) was used to measure the expression of selected AMPs in response to fungal infection. RT-qPCR reactions were performed in a final volume of 12 μL, containing: 6 μL of (2x) PowerUp™ SYBR™ Green Master Mix, 0.8 μM of primers to amplify amplicons corresponding to *cathepsin B*, *defensin A* and *cecropin*, 0.4 μM of primers for genes *actin* and *ADA-RP49*, 3 μL of cDNA [14]. The RT-qPCR conditions used were: 95 °C: 3 min, 40 cycles of 95 °C: 15 s, and 60 °C: 60 s, followed by a melt curve analysis to confirm the specificity of the reaction. In addition, the RT-qPCR reaction reproducibility was performed on three independent cDNA samples, and for each cDNA sample, the reaction was performed in triplicate (technical replicates). Relative differences in transcript levels were obtained using the ∆∆Ct method with *actin* and *ADA-RP49* as the reference genes [49, 50].

The efficiency of the *defensin A*, *cecropin, cathepsin B* and *actin* and *ADA-RP 49* primers was tested by serially diluting a cDNA sample with eight dilution points according to [51]. The concentrations were log-transformed and plotted on a linear regression slope (Additional file 2: Fig. S1). For primer sequences and details of PCR conditions, please see Additional file 2, Dataset S1 and Dataset S2, respectively.

### Statistical analyzes

The data obtained from hemocyte quantification, dynamics and PO activity were submitted to the Shapiro–Wilk normality test, analyzed by two-way ANOVA, followed by the Tukey test with a significance level of 5% (*P* ≤ 0.05). In addition, the RT-qPCR data were analyzed by one-way ANOVA followed by the Tukey test. All analyses were performed using GraphPad Prism v8.00, Inc (GraphPad Software, USA).

## Results

### The Midgut is a potential route of fungal infection

**Fig. 1:**
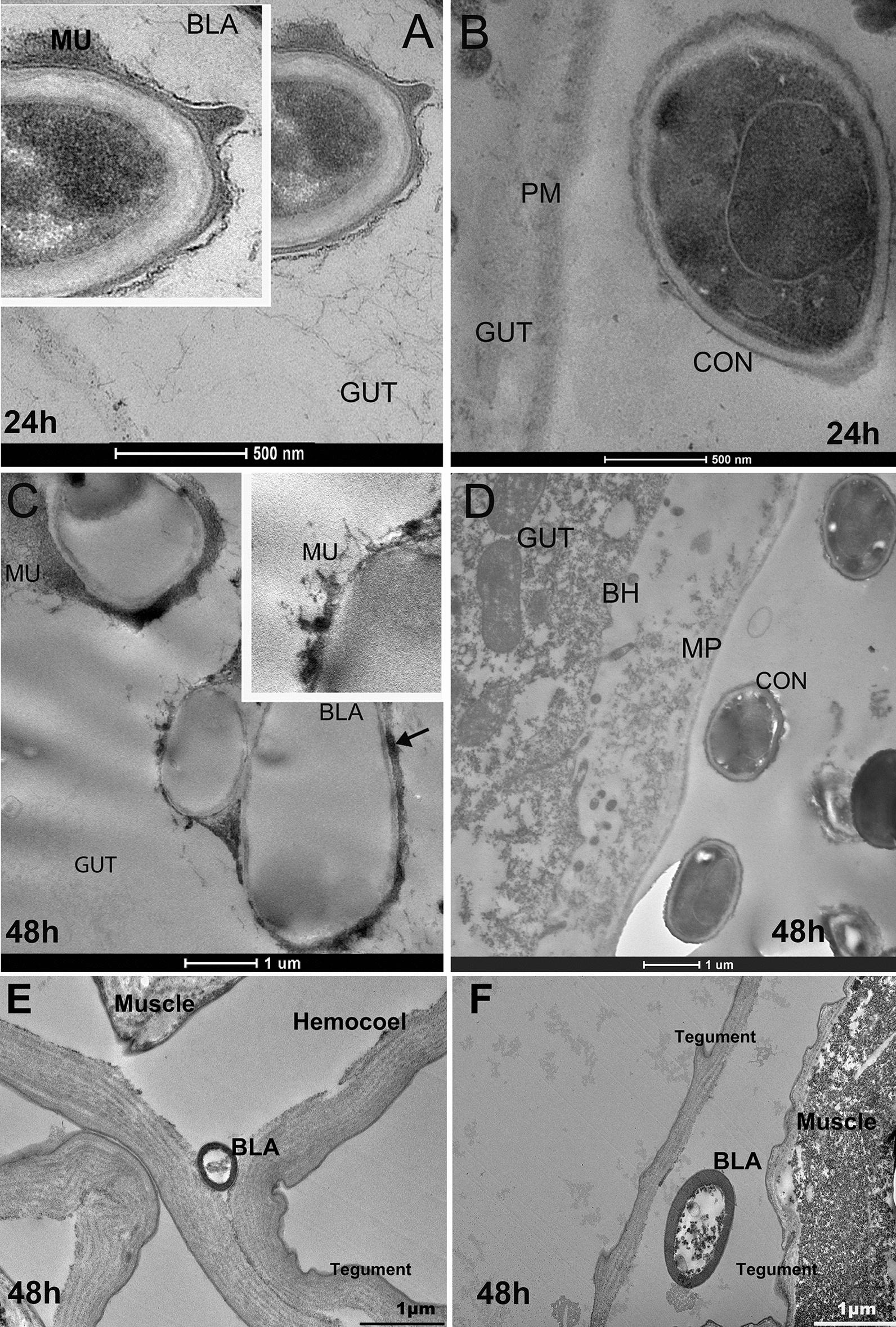
Photomicrography of blastospores (BLA) and conidia (CON) of *Beauveria bassiana* CG 206 infecting *Aedes aegypti* larvae at 24 (**A**, **B**) or 48 h (**C**–**F**) via the digestive tract (**A**–**D**) and hemocoel (**E**) and between 2 teguments (**F**). Material similar to mucilage (MU); or melanin (black arrow); peritrophic membrane (PM); brush border (BB)

Both propagules used the midgut as the main route of infection (Fig. 1A–D). In 24 and 48 h, blastospores produced an electron-dense material similar to mucilage (Fig. 1A, C). In addition, spots of electron-dense material that appeared similar to melanin were observed on blastospores within 48 h of infection (Fig. 1C-black arrow). Conidia were observed in the midgut but regardless of the time post exposure, conidia did not show activity such as germ tube development in the midgut (Fig. 1B, D). Although we did not see the propagules overcoming the enterocytes or tegument, at 48 h, we observed blastospores in the hemocoel of larvae exposed to blastospore suspensions (Fig. 1E, F).

### Quantification of hemocytes

**Fig. 2:**
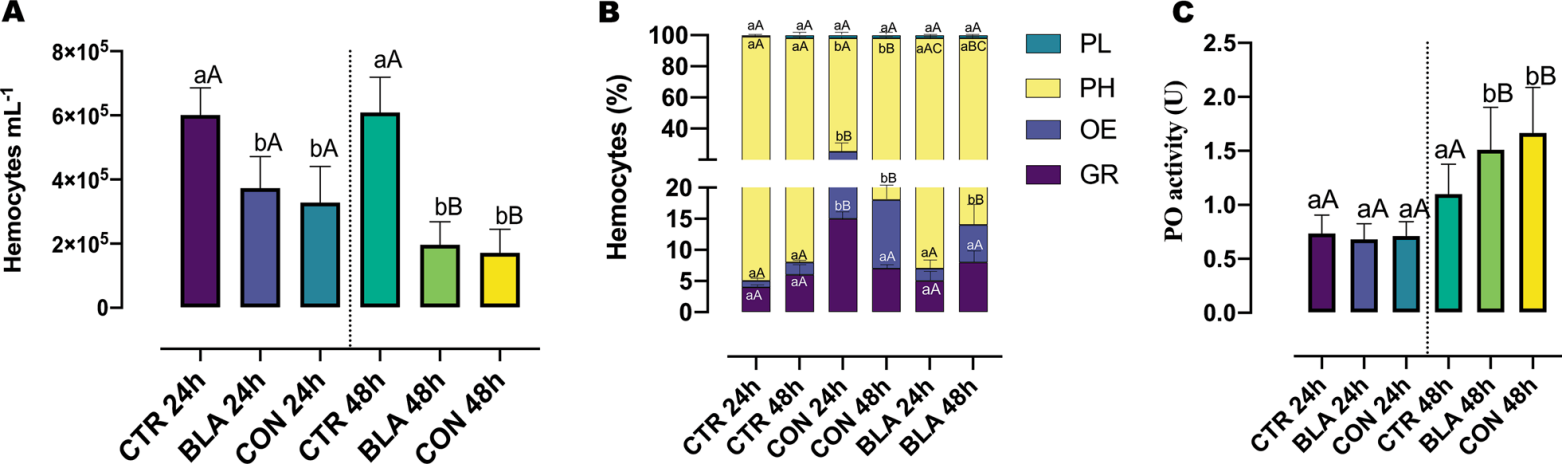
Total hemocytes concentration (**A**), the hemocyte dynamics (**B**) and phenoloxidase (PO) activity (**C**) in mosquito larvae exposed to blastospores (BLA) and conidia (CON) of *Beauveria bassiana* CG 206 for 24 or 48 h. Plasmatocytes (PL); prohemocytes (PH); oenocytoids (OE); granulocytes (GR). The same lowercase letters indicate no significant difference from each other (*P* < 0.05) at the same time comparing different groups. Identical capital letters do not differ (*P* ≤ 0.05) between 24 and 48 h in the same group

The total hemocyte concentrations (THC) were reduced by fungal infections (Fig. 2A). At 24 h p.e. to blastospores or conidia, the hemocyte concentrations were reduced (*P* = 0.0001; *P* < 0.0001, respectively) compared with the control group. Furthermore, both propagules showed similar results (*P* = 0.9054) in reducing the total hemocyte concentration.

After 48 h, the fungal propagules had a more significant impact in reducing the hemocyte concentrations (*P* < 0.0001) compared with the control groups (24 and 48 h) and the groups exposed to propagules for 24 h. Once again, exposure to blastospores and conidia had a similar effect (*P* = 0.9920) in reducing the hemocyte concentration.

### Population dynamics of hemocytes

Prohemocytes, granulocytes, oenocytoids, and plasmatocytes, were the main hemocytes observed and identified in the hemolymph. However, a few adipohemocytes and trombocytoids were also present (Additional file 1: Fig. S1). In larvae from the control group, prohemocytes were the main hemocytes observed, followed by granulocytes, oenocytoids, and plasmatocytes. Regardless of the treatment and time of exposure (24 or 48 h), the percentage of plasmatocytes was around 2% which was statistically similar (*P* > 0.9999) for all groups (Fig. 2B). At 24 h, the percentages of granulocytes were 4% (CTR), 5% (BLA), and 15% (CON); the oenocytoids percentage were 1% (CTR), 2% (BLA), 10% (CON), and the percentage of prohemocytes were 94% (CTR), 91% (BLA), 73% (CON) (Fig. 2B). After 24 h of exposure to conidia, the percentage of granulocytes and oenocytoids increased compared with blastospores (*P* = 0.0012; *P* = 0.0016, respectively) and the CTR (*P* = 0.0004; *P* = 0.0015, respectively). At the same time, the percentage of prohemocytes decreased in comparison to CTR and BLA (*P* = 0.0002; *P* = 0.0009, respectively).

By 48 h, the percentages of granulocytes were 6% (CTR), 8% (CON), and 7% (BLA); the oenocytoids percentages were 2% (CTR), 6% (BLA), 11% (CON), and the prohemocytes percentages were 90% (CTR), 84% (BLA) and 80% (CON) (Fig. 2B). Neither blastospores (*P* = 0.5325) nor conidia (*P* = 0.9550) affected the percentage of granulocytes compared with the control group. Conidia stimulated the increase in the percentage of oenocytoids compared with the control group (*P* = 0.0012) and blastospores (*P* = 0.0050) (Fig. 2B).

At 24 h, the percentage of granulocytes increased in larvae exposed to conidia compared with CTR 48 h (*P* = 0.0019). However, at 48 h, there was a significant reduction in the granulocyte percentage for the treatments with blastopores and conidia (*P* = 0.0238; *P* = 0.0061, respectively) compared with CON 24 h. In addition, larvae exposed to conidia for 24 h have a statistically higher percentage of oenocytoids than the CTR 48 h (*P* = 0.0018). Also, the oenocytoid percentage was statistically higher in larvae exposed to conidia for 48 h than in larvae exposed to the CTR 24 h (*P* = 0.0010) or the BLA 24 h (*P* = 0.0011) (Fig. 2B).

### Scanning electron microscopy of *Aedes aegypti* hemocytes

**Fig. 3:**
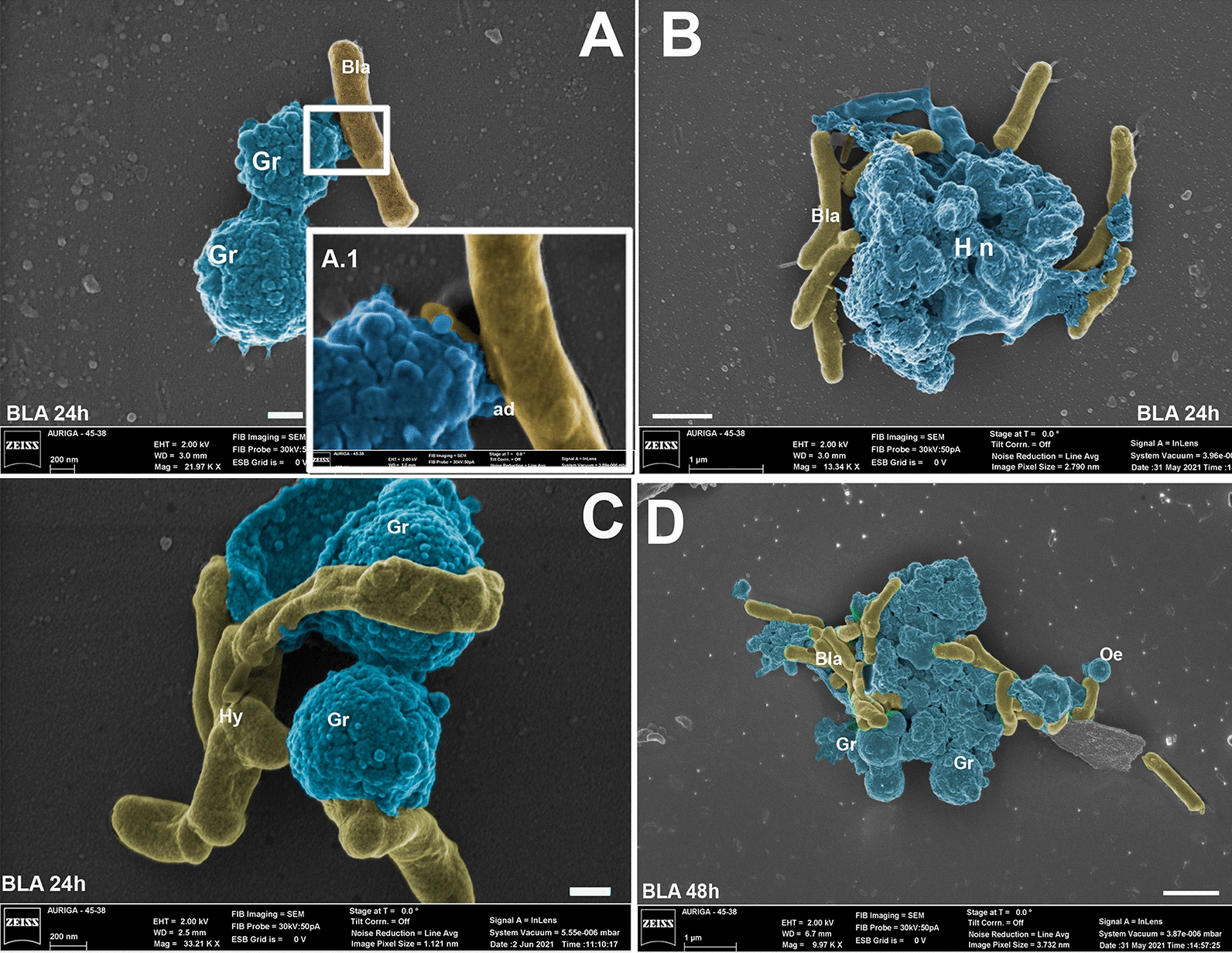
Photomicrography of hemocytes of *Aedes aegypti* larvae interacting with blastospores (BLA) and conidia (CON) of *Beauveria bassiana* CG 206 for 24 (**A**, **B**) or 48 h (**C**, **D**). Hemocytes nodulation process (Hn); granulocytes (Gr); Oenocytoids (Oe); F = fungus. Bars from figures **A** and **B** = 200 nm; bars from figures **C** and **D** = 1 µm

**Fig. 4:**
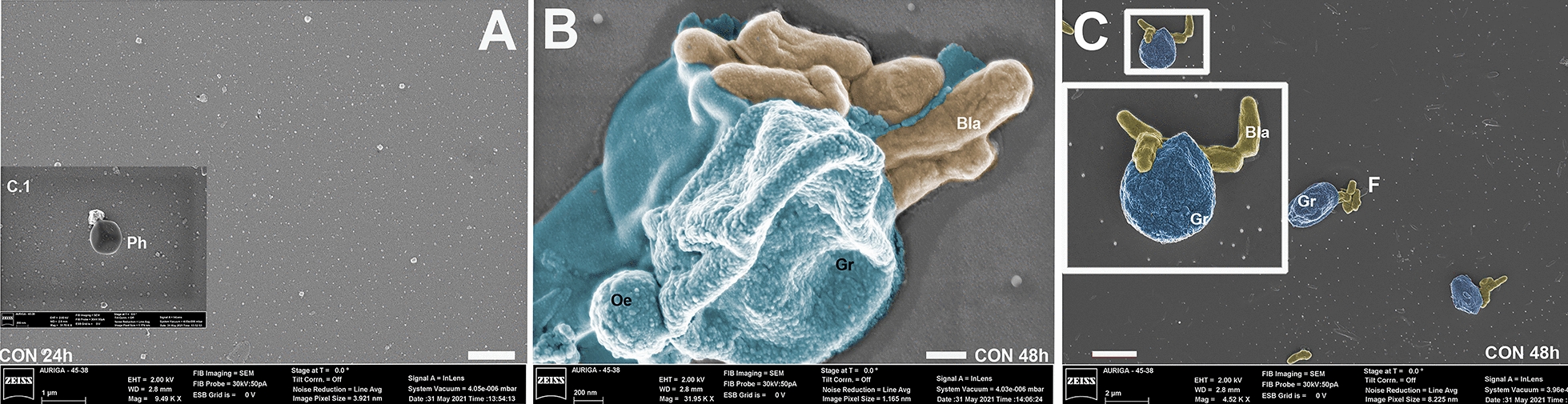
Photomicrography of hemocytes of *Aedes aegypti* larvae interacting with blastospores (BLA) and conidia (CON) of *Beauveria bassiana* CG 206 for 24 (**A**, **B**) or 48 h (**C**). Hemocytes nodulation process (Hn); granulocytes (Gr); Oenocytoids (Oe); plasmatocytes (Pl) F = fungus. Bars from figures **A** and **B** = 200 nm; bar from figure **C** = 1 µm

Scanning electron microscopy showed granulocytes, prohemocytes and oenocytoids (Figs. 3 and 4). Granulocytes exhibited a circular to oval shape with a granular surface (Figs. 3A, D and 4A, D). Oenocytoids are circular cells smaller than granulocytes, with a less granular surface (Figs. 3D and 4B).

Granulocytes and oenocytoids were seen to interact with blastospores (Figs. 3 and 4). For the larvae treated with blastospores, at 24 h, granulocytes adhered to the blastospores’ surface (Fig. 3A and A.1). At 24 h and 48 h, agglomerated hemocytes began to nodulate the blastospores (Fig. 3B, D). Also, at 24 h, granulocytes were nodulating hyphae (Fig. 3C).

For the larvae treated with conidia, no presence of the fungus was seen at 24 h, and the only hemocytes observed were prohemocytes (Fig. 4A). However, at 48 h, granulocytes and oenocytoids were seen nodulating blastospores (Fig. 4B and C).

### Measurement of PO activity in larvae exposed to fungus

At 24 h p.e., regardless of the propagule to which the larvae were exposed, their PO activity was not statistically different (*P* > 0.05) from the controls (Fig. 2C). By 48 h, the PO activity increased in larvae exposed to blastospores (*P* = 0.0343) and conidia (*P* = 0.0014) compared with the controls (Fig. 2C). However, also at 48 h, blastospores and conidia had similar results compared with each other (*P* = 0.8376). By 48 h, the PO activity increased (*P* < 0.0001) in larvae exposed to blastospores and conidia compared to the same propagules and the control group at 24 h.

### Differential expression of AMPs in larvae exposed to fungus

**Fig. 5:**
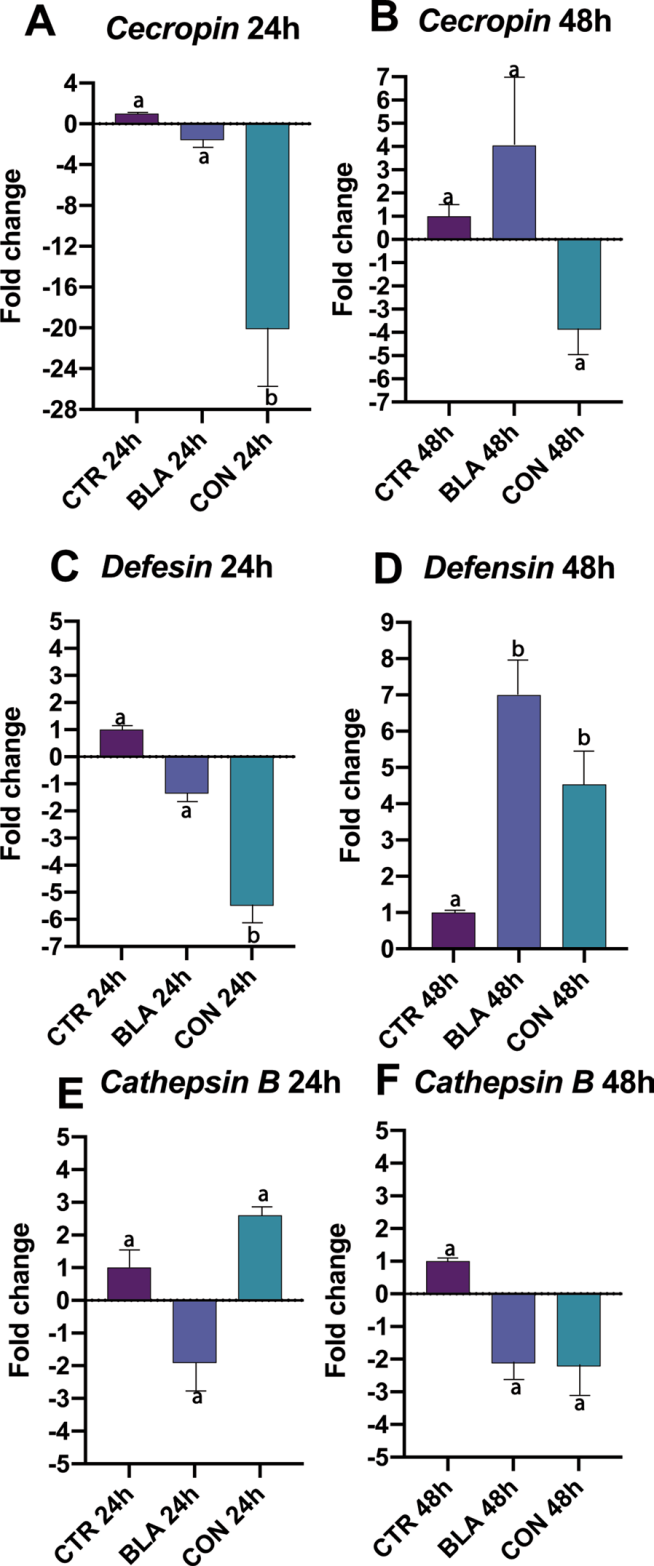
Antimicrobial peptides *cecropin*, *defensin A* and *cathepsin B* in *Aedes aegypti* larvae exposed to blastospores (BLA) and conidia (CON) of *Beauveria bassiana* CG 206 for 24 or 48 h. Equal letters do not differ (*P* ≤ 0.05) between 24 and 48 h in the same group

The levels of AMP transcripts in fungus-exposed larvae were compared with the levels expressed in larvae exposed to Tween 80 (control group). At 24 h there were a 20-fold and 18.6-fold downregulation in *cecropin* in larvae exposed to conidia compared with the control group (*P* = 0.0078) and blastospores (*P* = 0.0162), respectively. Furthermore, the relative expression of *cecropin* was similar between blastospores and the control group (*P* = 0.7777) (Fig. 5A). At 48 h, *cecropin* was downregulated by exposure to conidia (− 3.8 fold change) and was upregulated (+ fourfold change) by exposure to blastospores (Fig. 5B). Also, at 48 h, the *cecropin* levels were statistically similar (*P* > 0.05) for both propagules when compared with each other (i.e., BLA vs CON).

At 24 h, *defensin A* was significantly downregulated (-5.5 fold change, *P* = 0.0155) only in larvae exposed to conidia compared with the control group or the group exposed to blastospores (Fig. 5C). Furthermore, the expression of *defensin A* was statistically different in larvae exposed to conidia compared with the blastospores (*P* = 0.0161) (Fig. 5C). At 48 h, the relative expression of *defensin A* increased significantly in larvae exposed to blastospores (+ 7.1 fold change; *P* = 0.0086) and conidia (+ 4.5 fold change; *P* = 0.0198) compared with the control group (Fig. 5D). The transcription levels of *defensin A* were not statistically different when BLA vs CON were compared (*P* > 0.05) (Fig. 5D).

At 24 h, the expression of *cathepsin B* was downregulated by blastospore exposure (− 1.91 fold change) and upregulated by conidia exposure (+ 2.6 fold change). However, it was not significantly different (*P* > 0.05) in comparison to the control group (Fig. 5E). Finally, exposure to both propagules downregulated the expression of *cathepsin B* by 48 h. Again the *cathepsin B* expression was not significantly different (*P* > 0.05) regardless of treatment (Fig. 5F).

## Discussion

Our study provides new insights into EPF infections and interactions with the innate immune responses of *Ae. aegypti*. The cellular immune response, PPO cascade, and AMP expression play crucial roles in protecting insects against infections with filamentous fungi [35, 52, 53]. These data will help elucidate how each propagule interacts with, and modulate, immune pathways in *Ae. aegypti* larvae. In our previously study we evaluated the effects of *B. bassiana* at 10^5^, 10^6^, 10^7^, 10^8^ propagules mL^−1^ on against *Ae. aegypti* larvae. Both blastospores and conidia of *B. bassiana* CG 206 at 10^7^ propagules mL^−1^ have great potential to kill *Ae. aegypti* larvae and both used the digestive tract as a route of infection [10]. Thus, both propagules of *B. bassiana* CG 206 at 10^7^ propagules mL^−1^ were chosen for these assays.

In larvae treated with blastospores, hyphal bodies were observed in the hemocoel, and it was assumed that the fungus moved through the GI tract. Blastospores in the midgut were surrounded by electron-dense material similar to mucilage, and spots of a material similar to melanin [13]. *Aedes aegypti* larvae have four larval stages [54], and a blastospore was detected between two teguments. Therefore, the propagule may invade the soft cuticle or tegument during molting.

Both propagules were found in the mosquito midgut and a few blastospores were observed inside the hemocoel. Butt et al. [55] observed conidia in the gut of *Ae. aegypti* larvae, with no evidence of them breaching the midgut wall. We did not detect propagules invading the hemocoel or penetrating the tegument of the larvae as has been reported previously [14]. As hyphal bodies were rarely observed inside the hemocoel, *B. bassiana* CG 206 may need more than 48 h to reach and proliferate in the hemocoel. However, *B. bassiana* CG 206 does invade the mosquito midgut and reduces larval survival [10].

The total hemocyte concentration was reduced after 24 h of fungal exposure and this reduction was more prominent at 48 h. Although the fungus did not proliferate abundantly in the hemolymph, we suggest its presence in the gut may have stimulated hemocyte differentiation. Freitas et al. [56] observed a similar decrease in THC only 48 h after inoculating conidia of *B. bassiana* CG 206 into the tick, *Rhipicephalus microplus*. Wang et al. [57] exposed *Spodoptera litura* larvae to *Metarhizium rileyi* conidia by injection or immersion. They observed that the reduction in hemocyte concentration was slower in larvae immersed (~ 20 h) than injected (~ 2 h). We observed a decrease in THC at 24 h, suggesting that fungus might rapidly infect larvae after the immersion protocol.

Although a decrease in THC was observed, the granulocyte and oenocytoid percentages increased. Guimarães et al. [58] reported similar results in the sugarcane borer (*Diatraea flavipennella*) exposed to EPF. Mishra et al. [59] observed the opposite in larvae of *Musca domestica* L. exposed to *B. bassiana* (i.e., THC increase, granulocyte reduction, and similarity for oenocytoids). Here, the fungal infection did not affect the plasmatocyte percentage, corroborating other studies [58, 59]. Thus, we suggest that plasmatocytes do not play a pivotal role in *Ae. aegypti* larvae against *B. bassiana* CG 206. For conidia treatment, the prohemocytes percentage decreased at 24 h and increased by 48 h. Rodrigues et al. [60] observed similar results in *Anopheles gambiae* challenged with *Plasmodium sp*. Since prohemocytes are precursors of other hemocytes [61], this drop in percentage may be due to their differentiation in the first 24 h.

By 24 h after exposure to conidia, the numbers of granulocytes and oenocytoids increased more than when the larvae were exposed to blastospores. Blastospores are more efficient in not being detected by hemocytes since conidia have more Pathogen Associated molecular Patterns (PAMPS) such as *β*-glucan and chitin [16], which explains our results. Depending on the fungal isolate and host, conidia can rapidly turn into hyphal bodies (blastospores), deploy toxins to delay the host immune response and progressively colonize the host [17, 62]. However, by 48 h, the conidia may transform into hyphae [57], stimulating the cellular responses and recruiting prohemocytes to differentiate into granulocytes and oenocytoids. In addition, secondary metabolites such as beauvericin and destruxin might immuno-suppress the insects [63]. Fan et al. [64] observed that destruxins cause apoptosis of hemocytes. Although we did not conduct experiments involving toxins, the fungus might deploy secondary metabolites during the invading process, which could explain our results involving THC and hemocyte dynamics; however, further studies need to be conducted. These applications must consider the fungal isolate and concentration used, the application protocol, the application site, the target host, and the time of analysis.

Both propagules were observed in the midgut. The presence of conidia in the midgut might stimulate and recruit oenocytoids and granulocytes to act at the beginning of the infection, secreting AMPs into the gut lumen [16, 30, 61]. Meanwhile, blastospores were also observed surrounded by mucilaginous material, as observed by Alkhaibari et al. [13], which might delay the ability of the immune response to detect the threat.

By 48 h exposure to blastospores, dark spots of a material similar to melanin were observed, as described by Alkaibari et al. [13]. In addition, the PO activity increased only at 48 h regardless of the propagule. Interestingly, at 24 h, only conidia stimulated the oenocytoid recruitment. However, at 48 h, the percentage of oenocytoids also increased in larvae exposed to blastospores. These data reinforce our suggestion of a delay in the hemocyte response to blastospores. Also, we did not observe material similar to melanin surrounding conidia. Oenocytoids and enterocytes play a crucial role in releasing PO [65]. That functions in producing melanin [16, 66]. Blandin et al. [67] observed hemocytes delivering TEP 1 protein (involved in melanization) in the gut lumen of *A. gambiae* infected with *Plasmodium sp.* In addition, Shao et al. [68] reported the ability of enterocytes to melanize *Plasmodium* ookinetes.

Granulocytes are involved in encapsulating microorganisms [69]. Here, by 24 h, conidia stimulate the increase of granulocyte percentage, whereas blastospores take 48 h. These results suggest that the conidium is the first propagule detected by the immune response, even in the gut. Furthermore, by 24 h, we observed blastospores and hyphae directly interacting with hemocytes in larvae exposed to blastospores. However, for the treatment with conidia, this interaction between blastospores and hemocytes was observed only at 48 h. Although we did not observe the invasive process, these results reinforce the idea that the blastospores produced in vitro could quickly cross the midgut barrier and reach the hemocoel [15]

We observed hemocytes, mainly granulocytes and oenocytoids, adhered to the fungus, which demonstrated the involvement of both hemocytes to attempt to stop the fungal infection. It is the first time that SEM has shown this interaction. However, some researchers have used other techniques to report the fungus-hemocytes interaction, using a similar (i.e., *Aedes* vs. EPF) or another scientific model of study [36, 44, 70–73]

By 24 h, exposure to blastospores and conidia resulted in the downregulation of *cecropin* and *defensin A*, also observed by Alkhaibari et al. [15]. However, *cathepsin B* was slightly upregulated in larvae exposed to conidia. *Cecropin* and *defensin A* are likely transcribed de novo via the activation of the Toll pathway [33, 74, 75]. Cathepsins are serine proteases involved in an extensive range of biological processes, such as digestive and cell apoptosis, due to their proteolytic activity [76, 77]. These may be transcribed de novo or may be present before infection as inactive precursors that can be activated quickly to play a role in immunity. Caicedo et al. [79] reported the involvement of *Cathepsin B* blocking dengue virus invasion of the *Ae. aegypti* midgut. In addition, Lowenberger et al. [80] reported the involvement of the *Ae. aegypti* midgut in delivering defensins against *Plasmodium* sp*.* infection. Since conidia were observed in the midgut, we suggest that AMPs are secreted into the midgut lumen. However, fungal toxins such as destruxins might decrease AMPs transcription [81]. Therefore, as long as the fungus proliferates, the mosquito immunological system may improve its ability to recognize fungal propagules.

Interestingly, at 48 h, *defensin A* was remarkably upregulated, demonstrating that this AMP is the primary transcript against *B. bassiana* CG 206. In addition, we observed similar results involving the relative expression of *defensin A* in *Ae. aegypti* larvae exposed to *M. anisopliae* (unpublished data).

Filamentous fungi also can alter the gut microbiota balance of mosquitoes [36, 82]. It is possible that *B. bassiana* infections might also modulate the intestinal flora, affecting the larval immune system.

## Conclusion

*Beauveria bassiana* CG 206 isolate had different ways of infecting *Ae. aegypti* larvae, leading to theirdeath. The fungus interacted with mosquito larvae mainly by the midgut. The presence of fungal propagules stimulated the cellular response, PO activity, and AMP expression. However, these responses may take time to become activated, which allows the fungus a window of time to incapacitate the larvae and downregulate their immune responses. As well as demonstrating the ability of this fungal isolate to serve as a new biological control agent of mosquitoes, this study contributes to the growing literature to help us better understand the intricate molecular and immunological interactions between fungal pathogens and *Ae. aegypti* larvae.

## Supplementary Information

**Additional file 1: Text S1**. Protocol of insect hemolymph collection. **Figure S1**. Hemocytes identification (DIC and GIEMSA staining). **Figure S2.** Hemocytes identification (Scanning electron microscopy).

**Additional file 2: Dataset S1.** Real-time PCR conditions. **Datase S2.** Primers sequence. **Figure S1.** Primers efficiency.

## Data Availability

The datasets supporting the conclusions of this article are included within the article.

## Abbreviations

EPF: Entomopathogenic fungi
PO: Phenoloxidase
THC: Total hemocyte concentration
APMs: Antimicrobial peptides
GRAM +: Gram-positive bacteria
Gram −: Gram-negative bacteria
PPO: Prophenoloxidase
RH: Relative humidity
RT: Reverse transcription
DEPC: Nuclease-free water
