# Correction: *Beauveria bassiana* interacts with gut and hemocytes to manipulate *Aedes aegypti* immunity

**DOI:** 10.1186/s13071-023-05697-1

**Published:** 2023-03-02

**Authors:** Ricardo de Oliveira Barbosa Bitencourt, Thaís Almeida Corrêa, Jacenir Santos-Mallet, Huarrisson Azevedo Santos, Carl Lowenberger, Haika Victória Sales Moreira, Patrícia Silva Gôlo, Vânia Rita Elias Pinheiro Bittencourt, Isabele da Costa Angelo

**Affiliations:** 1 Graduate Program in Veterinary Sciences, Veterinary Institute, Federal Rural University of Rio de Janeiro, Seropédica, RJ Brazil; 2 Oswaldo Cruz Foundation, IOC-FIOCRUZ-RJ, Rio de Janeiro, RJ Brazil; 3 Centre for Cell Biology, Development and Disease, Department of Biological Sciences, Simon Fraser University, Burnaby, BC V5A 1S6 Canada; 4 Department of Animal Parasitology, Veterinary Institute, Federal Rural University of Rio de Janeiro, Seropédica, RJ Brazil; 5 Department of Epidemiology and Public Health, Veterinary Institute, Federal Rural University of Rio de Janeiro, Seropédica, RJ Brazil; 6 FIOCRUZ-PI, Teresina, Piauí Brazil; 7 Iguaçu University-UNIG, Nova Iguaçu, RJ Brazil

**Correction: Parasites & Vectors (2023) 16:17** **https://doi.org/10.1186/s13071-023-05655-x**

Following publication of the original article [1], the authors flagged that the name of the fourth author, Huarrisson Azevedo Santos, had been incorrectly spelled with 'Huarrison' (i.e., with just one 's'). The published article has since been updated to correct the name, and the corrected name may be seen in this erratum.
