# Supplementary material for: Beauveria bassiana interacts with gut and hemocytes to manipulate Aedes aegypti immunity
Source: Parasit Vectors. 2023 Jan 17;16:17. doi: 10.1186/s13071-023-05655-x (PMC9847134; doi:10.1186/s13071-023-05655-x)
Supplement: Supplementary file 1 — Additional file 1: Text S1. Protocol of insect hemolymph collection. Figure S1. Hemocytes identification (DIC and GIEMSA staining). Figure S2. Hemocytes identification (Scanning electron microscopy). [file 13071_2023_5655_MOESM1_ESM.docx]

**SUPPORTING INFORMATION TEXT**

- - 1. **Hemolymph collection and hemocytes Identification (DIC, GIENSA and SEM)**

Larvae (L_2-3_) were briefly anaesthetised by the cold (-4°C) (± 3 min until they stopped moving), dried with a paper towel, and transferred to sterile dry Petri dishes. Larvae were beheaded with a sterile scalpel blade [1]. The pool of hemolymph was collected with a microcapillary tube (0.03 mm) coupled to a flexible rubber and then immediately inoculated onto a glass slide, followed by fixation in methanol PA (Sigma-Aldrich) and stained with Giemsa (Sigma-Aldrich), according to [2]. For Differential interference contrast (DIC) microscopy, the pool of hemolymph was collected and dried at room temperature, followed by methanol fixation for 3 minutes. The hemocytes were registered by a photography system (Olympus®, UC30) coupled to a microscope (Olympus^®^ BX51, Tokyo, Japan). The hemocytes identification were according to [3] and [4].


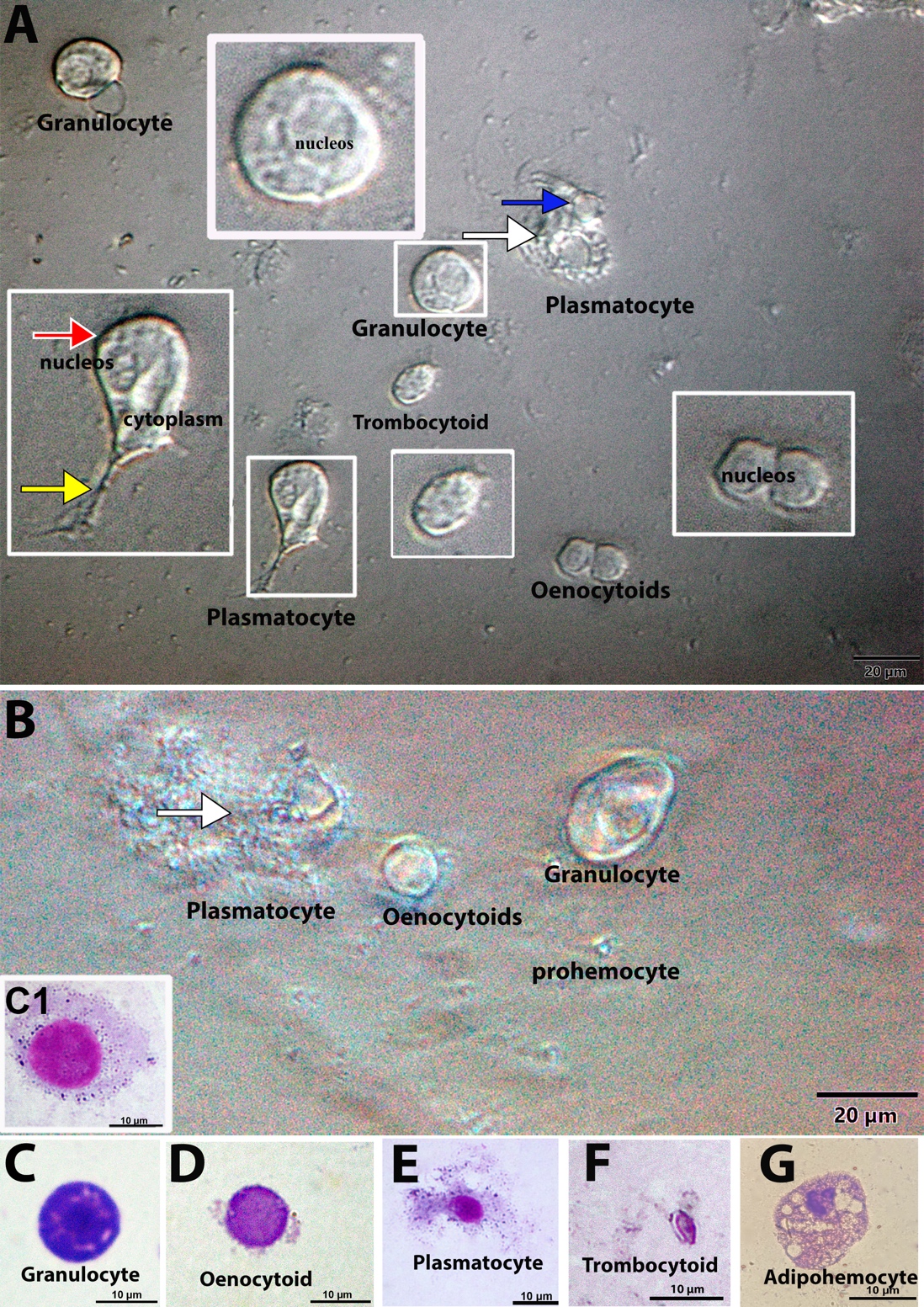


**Figure 1** Differential interference contrast (DIC) microscopy (A, B) and light microscopy (C1, C, D, E, F, G). Plasmatocytes exhibiting spindle-shaped (red arrow) to round (blue arrow) and eccentric nucleus and the cytoplasmatic membrane exhibited irregular filopodia (yellow arrow) or pseudopodia (white arrows); Granulocytes exhibiting fusiform or circular shape with central nucleus with (C1) or without pseudopodia (C). Granulocytes with black dots in the cytoplasm (C1); Oenocytoids are small than granulocytes with homogenous cytoplasm without pseudopodia (E); Trombocytoids with piriform shape (F); adipohemocytes exhibiting oval shape and cytoplasm with vesicles similar to lipid droplets (G).


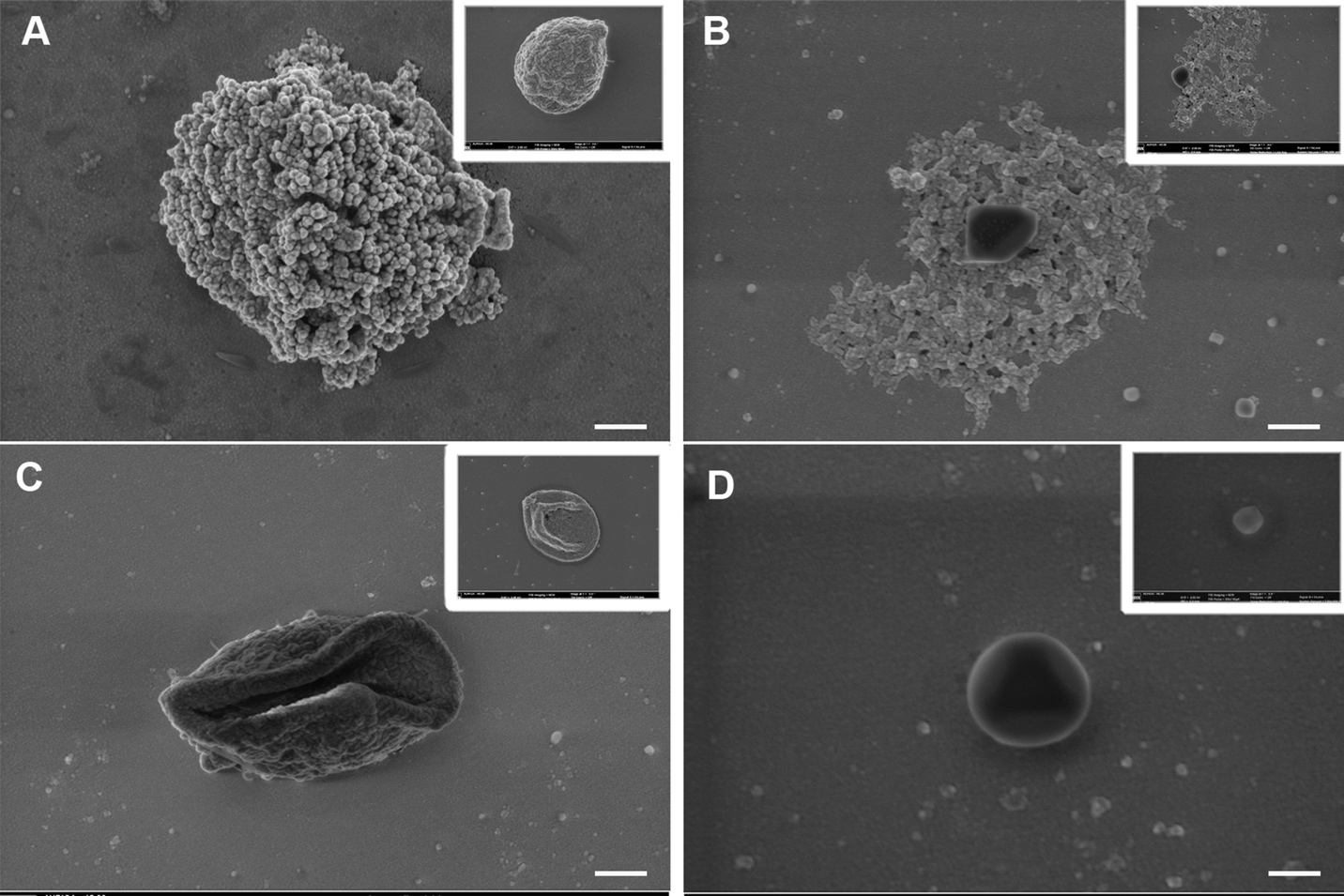


**Figure 2** Identification of hemocytes from Aedes aegypti larvae by scanning electron microscopy. A= granulocytes; B= plasmatocytes; C= oenocytoids; D=prohemocytes. White bar = 200 nm. Hemocytes showed the following characteristics: Spherical granulocytes with a granular or rough surface; plasmatocytes exhibiting projections attached to the surface; oenocytoids with a smooth surface and concave shape; prohemocyte with smooth surface and spherical shape.

1. **Supporting information references**
2. Nunes FC. Estudo da atividade larvicida de *Agave sisanala* contra *Aedes aegypti*. Universidade Federal da Paraíba. João Pessoa, Paraíba; 2013. p.114.
3. Fiorotti J, Menna-Barreto RFS, Gôlo PS, Coutinho-Rodrigues CJB, Bitencourt ROB, Spadacci-Morena DD, et al. Ultrastructural and cytotoxic effects of *Metarhizium robertsii* infection on *Rhipicephalus microplus* hemocytes. Front Physiol. 2019; 29:10:654.
4. Araújo HC, Cavalcanti MG, Santos SS, Alves LC, Brayner FA. Hemocytes ultrastructure of *Aedes aegypti* (Diptera: Culicidae). Micron. 2008;39:184–189.
5. Hillyer JF, Christensen BM. Characterization of hemocytes from the yellow fever mosquito, *Aedes aegypti*. Histochem Cell Biol. 2002; 117(5):431–40.
