# Supplementary material for: Beauveria bassiana interacts with gut and hemocytes to manipulate Aedes aegypti immunity
Source: Parasit Vectors. 2023 Jan 17;16:17. doi: 10.1186/s13071-023-05655-x (PMC9847134; doi:10.1186/s13071-023-05655-x)
Supplement: Supplementary file 2 — Additional file 2: Dataset S1. Real-time PCR conditions. Datase S2. Primers sequence. Figure S1. Primers efficiency. [file 13071_2023_5655_MOESM2_ESM.docx]

**SUPPORTING INFORMATION**

**Dataset S1**

1. **RT-qPCR conditions**

**Table 1** polymerase chain reaction condition.

| Primer | Concentration | Activation | Denaturation | | | Annealing |
| --- | --- | --- | --- | --- | --- | --- |
| *Cathepsin B* | 0.8µM | 95ºC (10”) | | 50ºC (15”) - 40cycles | 72ºC (20”) | |
| *Defensin A* | 0.8µM | 95ºC (10”) | | 61.5ºC (60”) 40 cycles | 72ºC (30”) | |
| *Cecropin* | 0.8µM | 95ºC (10”) | | 61.5ºC (60”) 40 cycles | 72ºC (30”) | |
| *Actin* | 0.4µM | 95ºC (10”) | | 61ºC (60”) 40 cycles | 72ºC (30”) | |
| *ADA-RP49* | 1 µM | 95ºC (10”) | | 61ºC (60”) 40ciclos | 72ºC (30”) | |

**2.1 Primers for RT-qPCR (Dataset S2)**

**Table 2** Primers forward and reverse for gene expression.

| Gene | GenBank access | Putative function | Forward (FW) and reverse (RV) primers | Amplicon (base pairs) | Reference |
| --- | --- | --- | --- | --- | --- |
| Cecropin | AY064080.1 | PAM | FW: ATGAACTTCACGAAGTTATTTCTC  RV: ACTTTCTTAGAGCTTTAGCCCC | 178 | [1] |
| Defensin A | AF156088.1 | PAM | FW: GCGACCTGCGATCTGCTGAG  RV: TCAATTTCGACAGACGCAGACC | 123 | [1] |
| Cathepsin b | AAEL007585 | PAM | FW: CAGGGATGTCACCCATATC  RV: GAAAGCAGCCTGAACTGG | 210 | [2] |
| Actin | NM_001101 | Housekeeping | FW: ATTAAGGAGAAGCTGTGCTACGTC  RV: CATACGATCAGCA TTACCTGGG | 158 | [3] |
| Ribosomal protein 49 (ADR-P49) | AAEL003396 | Housekeeping | FW: ACAAGCTTGCCCCCAACT  RV: CCGTAACCGATGTTTGGC | 97 | [3] |

PAM=antimicrobial peptide

1. **Primer efficiency**


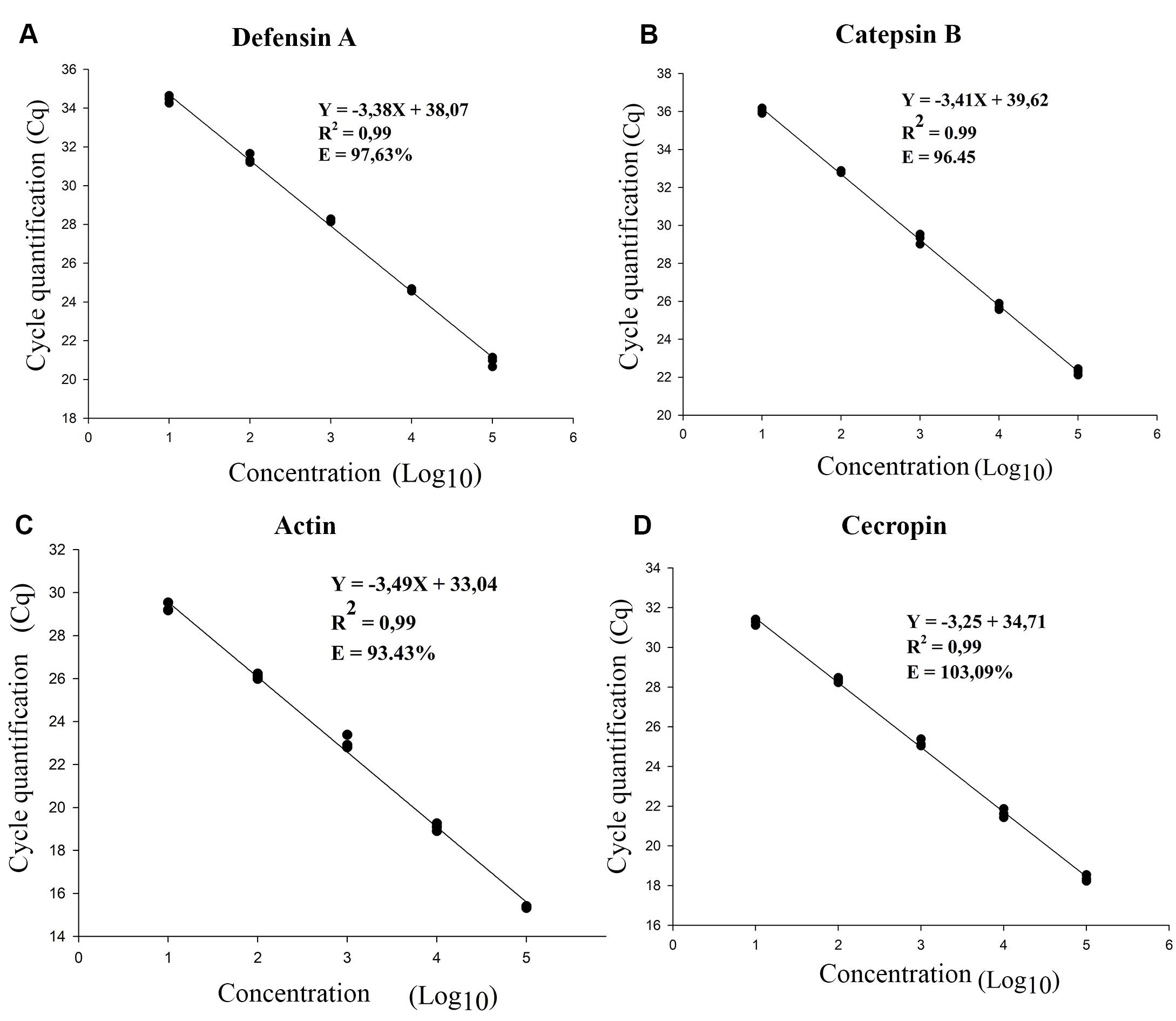


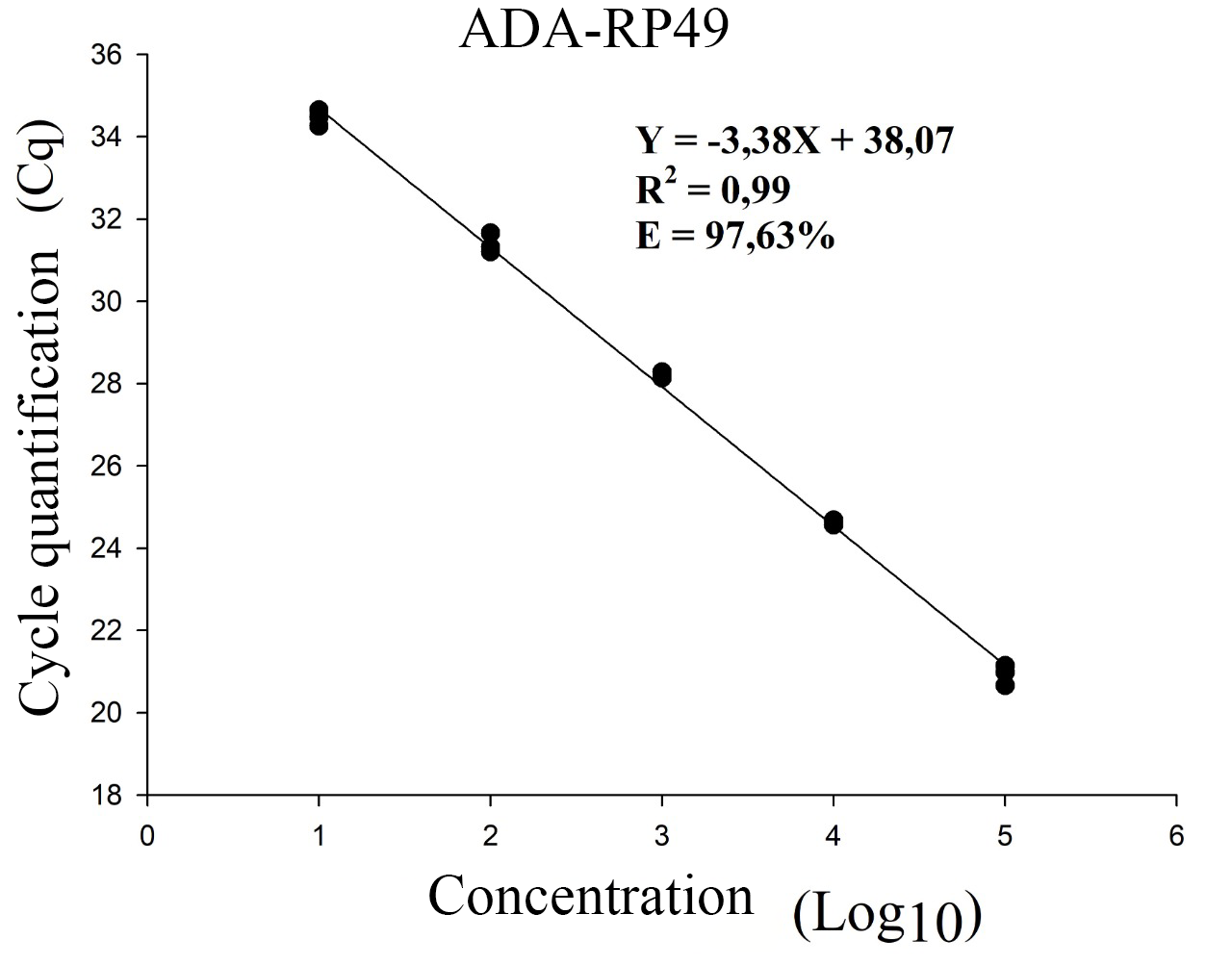


**Figure 1** Defensin A, cathepsin B, cecropin, actin, ADA-RP49 efficiency curves. The following formula was used to calculate the efficiency: PCR efficiency (%) = 10(^-1/5^) -1) × 100.

1. **Supporting information references**
2. Cooper DM, Chamberlain CM, Lowenberger C. *Aedes* FADD: a novel death domain-containing protein required for antibacterial immunity in the yellow fever mosquito, *Aedes aegypti*. Insect Biochem Mol Biol. 2009; 39(1):47–54.
3. Caicedo PA, Serrato IM, Sim S, Dimopoulos G, Coatsworth H, Lowenberger C, et al. Immune response-related genes associated to blocking midgut dengue virus infection in *Aedes aegypti* strains that differ in susceptibility. Insect Sci. 2019;26:635–648.
4. Alkhaibari AM, Carolino AT, Yavasoglu SI, Maffeis T, James C B, et al. *Metarhizium brunneum* blastospore pathogenesis in *Aedes aegypti* larvae: attack on several fronts accelerates mortality. PLoS Pathog. 2016;12:1–19.
